# Prediction of Protein–Protein Interactions by Evidence Combining Methods

**DOI:** 10.3390/ijms17111946

**Published:** 2016-11-22

**Authors:** Ji-Wei Chang, Yan-Qing Zhou, Muhammad Tahir Ul Qamar, Ling-Ling Chen, Yu-Duan Ding

**Affiliations:** 1 National Key Laboratory of Crop Genetic Improvement, Huazhong Agricultural University, Wuhan 430070, China; longkaichang@163.com (J.-W.C.); zhyq2611@163.com (Y.-Q.Z.); m.tahirulqamar@hotmail.com (M.T.U.Q.); llchen@mail.hzau.edu.cn (L.-L.C.); 2 College of Informatics, Huazhong Agricultural University, Wuhan 430070, China

**Keywords:** interaction prediction, PPIs, physical interactions, support vector machine

## Abstract

Most cellular functions involve proteins’ features based on their physical interactions with other partner proteins. Sketching a map of protein–protein interactions (PPIs) is therefore an important inception step towards understanding the basics of cell functions. Several experimental techniques operating in vivo or in vitro have made significant contributions to screening a large number of protein interaction partners, especially high-throughput experimental methods. However, computational approaches for PPI predication supported by rapid accumulation of data generated from experimental techniques, 3D structure definitions, and genome sequencing have boosted the map sketching of PPIs. In this review, we shed light on in silico PPI prediction methods that integrate evidence from multiple sources, including evolutionary relationship, function annotation, sequence/structure features, network topology and text mining. These methods are developed for integration of multi-dimensional evidence, for designing the strategies to predict novel interactions, and for making the results consistent with the increase of prediction coverage and accuracy.

## 1. Introduction

**Figure 1:**
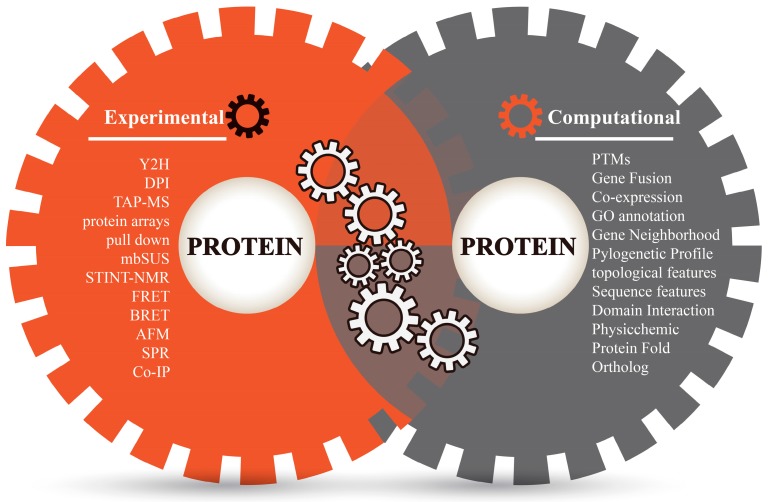
Different methods for detecting protein−protein interactions.

Proteins perform their complicated functions by physically interacting with other proteins. Sketching a map of protein–protein interactions (PPI) is a significant topic of system biology and an important step towards understanding protein functions and cellular behaviors [1]. Different experimental techniques (in vivo or in vitro) have made significant efforts to study the constant nature of protein interaction sites and screen a large number of protein interaction partners (Figure 1), such as two-hybrid (Y2H) screens, Tandem affinity purification mass spectroscopy (TAP-MS), protein microarrays, mating-based split-ubiquitin system (mbSUS), pulldown assays, dual polarization interferometry (DPI), NMR-based method for mapping the structural interactions (STINT-NMR), bioluminescence resonance energy transfer (BRET), fluorescence resonance energy transfer (FRET), atomic force microscopy (AFM), surface plasmon resonance (SPR), protein complex immune precipitation (Co-IP) [2,3,4,5], and so on. Among these experimental techniques, some high-throughput methods such as Y2H, TAP-MS, protein chips, etc. have been comprehensively applied to detect a protein’s binary interactions and to generate many genome-scale protein interaction networks in model organisms such as *Homo sapiens* [6], *Drosophila melanogaster* [7], *Saccharomyces cerevisiae* [8], and *Caenorhabditis elegans* [9]. However, genome-scale experiments are costly and labor-intensive, and have inherent biases and limited coverage. Limitations of equipment resolution and environmental disturbances during operations (such as purification, capture, equilibrium, signal label and imaging) could inevitably lead to errors and biases in experimental techniques [5,10]. Moreover, the potential of protein interactions within an organism is enormous; for example, total interactions of human PPIs are estimated to be over 650,000 [11]. As far as we know, experimental findings are often incomplete even for well-studied model organisms, not to mention other species. Therefore, the verification of a universal PPI network is a great challenge to laboratory work and necessitates more revolutionary technologies.

Bioinformatics techniques of PPI prediction strengthen and flourish the study of protein interactions (Figure 1). Bioinformatics approaches consider the term of “protein–protein interactions” as the associations between proteins that include relationship aspects of evolution, function and structure. These techniques overcome the limitations of experimental techniques, are beneficial to complete the missing pieces of experimental PPI data and help in discovering the clues of PPI mechanisms in silico. Up until now, several computational methods have been successfully applied to predict protein interactions in multiple perspectives: phylogenetic profile [12], protein sequence [13], domain–domain interaction (DDI) [14], coexpression [15], ortholog [16], etc. These methods are mainly focused on individual (or homogeneous) evidence for prediction and have certain specificities as well as biases [1,17]. An alternative strategy is the integration of evidence sources in a statistical learning framework. Combining evidence exhibits the strength of machine learning and data mining to overcome the limitations of independent predictions and make the results consistent with the increase of prediction coverage and accuracy [1,18,19,20,21,22,23]. Such methods of PPI prediction are referred as “prediction of protein–protein interactions by evidence-combining methods”.

In this review, the workflows for prediction pair-wise PPIs by combined evidence from studies building PPI networks on the genome scale level are presented and discussed. The presented workflows mainly consist of three basic steps: (1) Defining gold standard datasets/training datasets of interacting and non-interacting protein pairs; (2) Characterizing the interactions by annotating gold standard datasets with diverse and carefully chosen evidence; this is an encoding process to turn protein interaction features into machine-readable rules; (3) Determining the probability of particular interactions by individual evidence, and thus combining the probabilities (or encoded vector) of all evidences to uncover the novel subset of the interactome.

## 2. Defining Gold Standard Datasets

Units of gold standard datasets are usually constructed for training or testing of PPI prediction. Datasets for training and testing units are generally independent. The quality and reliability of gold standard datasets for training affect the performance of different machine learning methods [17].

**Figure 2:**
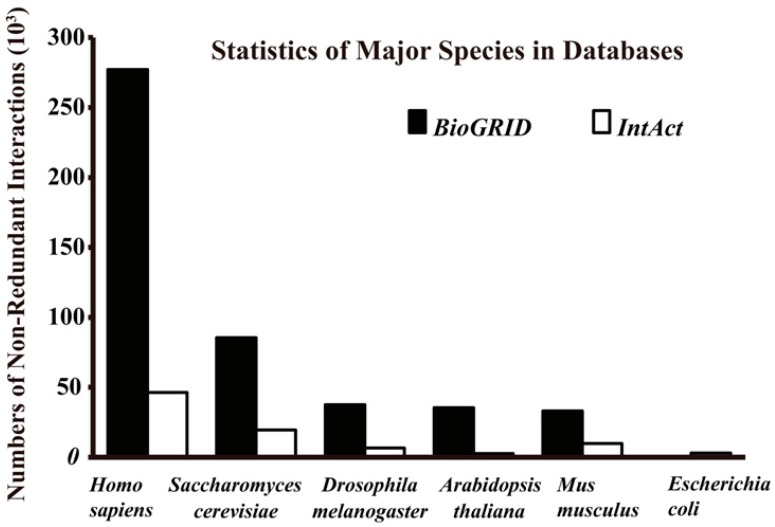
Total non-redundant interactions of major species in BioGRID (Version 3.4.140, September 2016) and IntAct (September 2016).

The gold standard positive (GSP) datasets are basically PPIs with high experimental confidence or reference evidence. Some of the datasets are available in public databases, such as: the Biological General Repository for Interaction Datasets (BioGRID) [24], the IntAct molecular interaction database (IntAct) [25], Search Tool for the Retrieval of Interacting Genes (STRING) [26], Agile Protein Interactomes DataServer (APID) [27], the Database of Interacting Proteins (DIP) [28], HitPredict [29], the Molecular INTeraction database (MINT) [30], the Arabidopsis Information Resource (TAIR) [31], the Human Protein Reference Database (HPRD) [32], Protein Interaction Network Analysis (PINA) platform [33] and the High-quality INTeractomes database (HINT) [34]. These repositories of protein complexes and interactions are varied in size and species-specificity, and they contain information from experimental and computational sources with or without manual validation (Table 1). For these reasons, it is advised to choose high-quality positive datasets from multiple (times or methods) independent assays (usually high-throughput methods that consider the coverage and biases of different assays) [1] or from text mining of published literature with careful evaluation [2]. The gold standard datasets are always focused on reference datasets that source from model organisms (Figure 2) with advanced accuracy and coverage. This repository is very helpful for seeking out general clues of PPI mechanisms in silico, and supporting studies which lack the existing data of a targeted organism [1,16,35]. However, it is also a double-edged sword that inevitably leads to errors and biases by over-fitting of specific data in the minority organisms.

Gold standard negative (GSN) datasets generally cannot be obtained by direct experimental measures. There is a Negatome database (2.0) [37] which provides a collection of protein and domain pairs unlikely to be engaged in direct physical interactions (supported by text mining and 3D structure of protein complexes) (Table 1). Unfortunately, due to the limited data (about 6000 pairs at present), this non-interacting dataset could not satisfy the diverse GSP datasets of different users. There are some reported methods for extracting negative datasets, such as: (1) Negative datasets are constructed by using random pairs which exclude the experimentally detected interactions [1], and as there are discordant numbers between high-confidence interactions and random pairs, the scale and structure of networks should be balanced between negative and positive datasets. This method may include undetected PPIs; (2) Negative examples are chosen based on the categories of their distinct functions, such as sub-cellular localization (can be accessed by tools such as LOCATE [38], PSORTdb 3.0 [39], LocDB [40]) and annotations (such as KEGG pathways, gene ontology (GO), and Enzyme Commission (EC)) [22,41]. However, these methods can also lead to biases due to varying definitions of categories [42]; (3) Another alternative approach is based on topological policy: choose pairs of separated proteins in existing PPI networks to represent non-interactions: defining negative samples as the protein pairs with the shortest path lengths exceed the median shortest paths in a GSP network [43], or further construct a GSN network based on the principle of keeping the composition and degree of a node identical to the GSP network [20]. The negative samples, however, still contain biases if the referential networks are partial [17].

## 3. Annotate Protein Pairs with Diverse Evidence

The characterization of existing interactions is usually processed to explore the crucial role of protein interactions. Interactions can convert proteins/polypeptides into transient or permanent complexes and the binding is determined by different elements such as cell physiology (function switches, regulation status, etc.), biochemistry environment (ions, dipoles, Van der Waals forces, etc.) and shape of the binding surface (3D structure, folding elements, amino acid composition, etc.), which are further involved in the fields of functional genomes, dynamics, kinetics, mechanics etc. [3,4,44]. Experiments for detecting PPIs in vivo and in vitro are aimed at capturing and displaying the specific nature of protein interactions under a certain condition. However, the strategies of prediction of PPIs in silico are devoted to extracting machine-learned PPI rules (usually unintelligible to humans) from interaction-related features and are used to predict unexploited PPIs. Evidence for machine learning includes physical features (such as calculated statistics of hydrophobicity, hydrophilicity, polarizability, etc.) and non-physical features (such as gene coexpression, sequence similarity, function annotation enrichment, etc.). Each feature provides a different angle to view protein interactions and has the potential for uncovering a novel subset of the whole interactome. For this reason, during the workflow of PPI prediction, protein pairs are generally annotated by different parameters (individual or co-occurring parameters) taken from diverse sources of evidence, such as evolutionary relationship, functional annotation, sequence/structure features, network topology and text mining (Table 2).

Evolutionary Relationship: Methods based on evolutionary information use genomic context of organisms to infer functional associations between proteins, including gene neighborhood [70], gene fusion [71] and phylogenetic profiles [12]. (1) The basic hypothesis of the gene neighborhood method is that if neighbor associations of multiple genes are conservative across genomes, it infers that those genes/proteins may have function association which implies interactions; (2) Gene fusion events are also called the “Rosetta stone” method. It is based on the hypothesis that the homology of two interactive proteins/domains in one species may fuse into a single protein in another species. Generally, organisms’ sequences are compared to detect the Rosetta stone (domains) fusion events in selected organisms. The fusion phenomenon indicates the functional association and possibility of forming a protein complex; (3) Phylogenetic profile hypothesized that functionally linked proteins tended to coexist during evolution, and the two proteins with similar profiles (inherited together) in different species might have interactions or functional linkages. Sequence comparisons between genomes are used to construct phylogenetic profiles (A protein/domain is represented as an *N*-dimensional vector: N, number of genomes; Value = 1 or 0, presence or absence of protein/domain in an organism) and evaluate protein pairs by measuring distance.

Ortholog: If a pair of proteins has high similarity to the sequences of another pair of genes or proteins with known interaction in other species (orthologous proteins), they are supposed to have similar functions which infers the relationship of interactions. This approach usually uses sequence alignment algorithms to define the similarity of full sequences or residues, which is regarded as an index to predict interactions between proteins [1,50,51].

Gene Function Annotations: This method is based on the hypothesis that two proteins functioning in the same biological process should be more likely to interact with each other than those two proteins not sharing the same biological process. Information of biological function is accessible from some hierarchically structured annotation systems, such as GO, KEGG, EC and MapMan (usually used for plants) [72], which provide information of colocalization and participation in a shared cellular process implicit to PPIs.

Coexpression: It is generally acknowledged that a pair of interacting proteins has relative gene expression, although the gene coexpression methods are an indirect way to infer the protein interaction (some results indicated that there is no straight correlation between gene expression profiles and PPI associations under some conditions [73]). However, gene co-expression contains information of transcription and regulation, and can be utilized to validate PPIs by calculating correlation coefficient of transcriptome data including RNA sequencing, DNA microarrays, expressed sequence tag (EST), etc. [1]. In addition, by applying the clustering algorithms or analyzing topological structure of coexpression network [73], cluster modules can help to reveal functional relationships and predict PPIs.

Sequence-Based Code Signatures: Some studies implement the natural language processing (NLP) technique to encode sequences for perdition of PPIs. The language of protein sequences is translated into sequence-based signatures and mapped into high-dimensional vectors by using the occurrence frequencies of each kind of building block [74]. Different signatures are wildly used, including N-grams, ORF codon, Conjoint Triad, etc. The “N-grams” (natural language processing term refers to N consecutive symbols) are sets of all possible subsequences of amino acids in protein sequences (N-grams: *N* = 3, total number = 8000 (20^3^)) [60,61]. ORF codon uses 64-dimensional vectors to represent a given open reading frame (ORF) instead of an amino acid [62].

**Figure 3:**
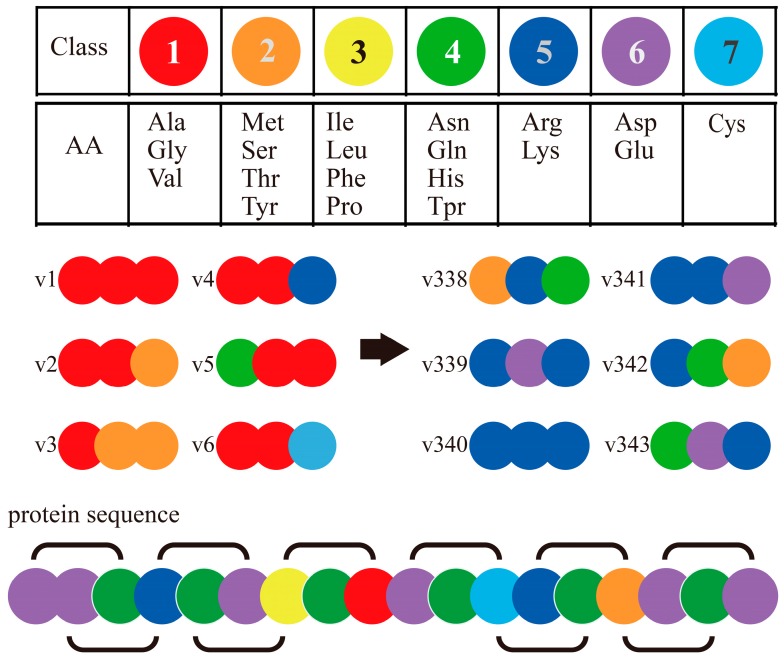
Schematic diagram for Conjoint Triad Method [55].

The Conjoint Triad Method (also called Shen’s method) [75] is one of the popular codon usage methods of sequence-based PPI prediction. It encodes each protein sequence as a feature vector by observing frequency of amino acid (AA) triads as follows (Figure 3): (1) It encodes/classifies 20 amino acids into seven classes based on their dipoles’ strength and volume of the side chains; (2) A protein sequence is resolved into a series of AA triads (three continuous AAs as a unit); (3) It uses 343 (7^3^)-dimensional vectors to represent a given protein, and each element of this vector is the frequency of an AA triad; (4) The PPI pair is represented by concatenating the individual two vectors of corresponding proteins. It is noticed that, if we do not process the AA cluster step (in step 1), protein pairs will be required to get encoded as a 16,000-dimensional vector (20^3^ × 2, as N-grams method), which is too large for most classifiers. The rule of seven classes for 20 AAs is effective and convenient to operate, and is developed as a classical method that has been widely applied in interaction prediction and interaction site prediction based on sequences [58].

Sequence-Based Structure Signatures: Structure and chemical properties of a protein sequence can be translated into structure signatures to represent characteristics of a residue interface. These signatures include: (1) Physicochemical properties of amino acids, such as hydrophobicity, hydrophilicity, polarizability, solvent-accessible surface area (SASA), relative surface accessibilities (RSA) of residues, side chain net charge index (NCI), charge, isoelectric point, etc.; (2) Signatures of protein structure, such as 3D structure indexes in PDB, protein fold alpha helices, beta sheets and coils, posttranslational modifications (PTMs), and domains [1,76]. These signatures are available from different tools, including NACCESS program [77], DSSP algorithm in PDB [78], PSIPRED [1], AA index [79], etc.

**Figure 4:**
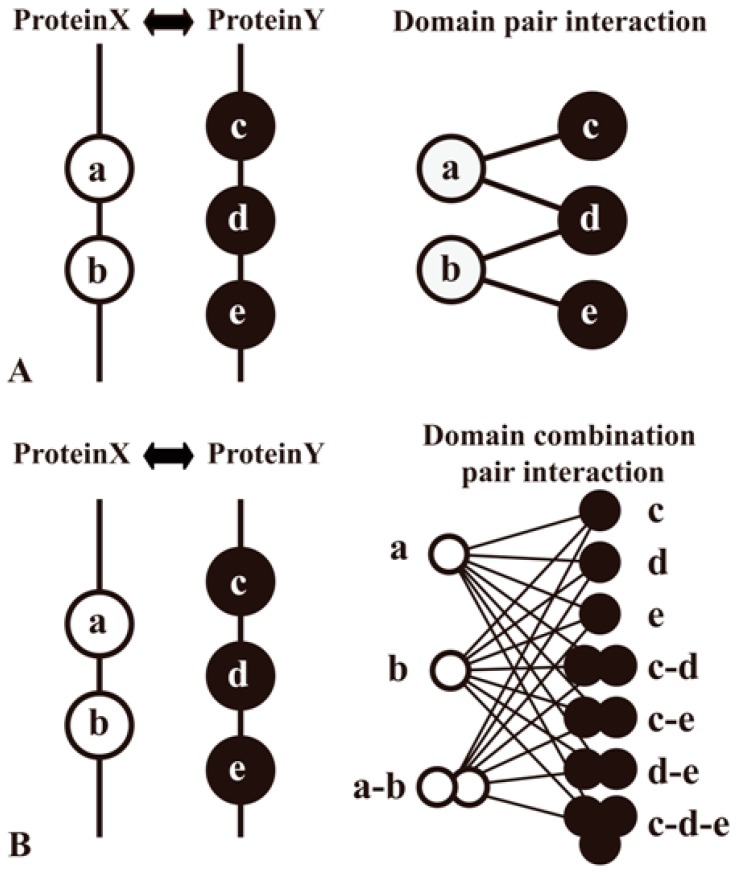
Two methods to predict domain−domain interactions (DDIs) from PPIs. Proteins A and B are a pair of proteins in a PPI network. Protein A contains domains a and b, whereas protein B contains domains c, d and e. PPI is interpreted as the result of interactions among multiple domain pairs. (**A**) A method that considers a domain pair as basic unit of protein interactions; (**B**) Another method that proposes a domain combination pair as a basic unit for the prediction model [83].

Domain methods aim to establish protein relationships by domain−domain interactions (DDIs), which are applied widely in sequence-based PPI prediction [35,45,46,47,48] As the domains are conserved, distinct, compact structural units in proteins, the computational insights into detailed knowledge about a protein pair’s interaction can be typically simplified as domain associations. Information of protein domains can be accessed at Pfam [80], Conserved Domain Database (CDD) [81], etc. Large-scale inference of DDIs can be processed by analyzing the domain composition of a protein pair in a high-quality PPI network and then using specific classifiers to identify domains (or domain combinations) responsible for protein interactions (Figure 4). Moreover, some prediction work of DDIs complements other evidence. For example, the DOMINE database [46,82] integrates other evidence for DDI inferences, such as phylogenetic profile, gene fusion, GO, etc.

Network Topology: Network topological parameters are generally calculated from positive datasets. They characterize the topological properties of currently available protein interaction networks to evaluate target protein pairs. Graph-theoretic invariants include weighted domination number, average eccentricity number, the eccentricity, circumference, weighted peripheral number, clustering coefficient of a protein pair, etc. [57].

Text Mining: Protein–protein interactions can also be predicted using text mining (TM). TM technology could explore protein interactions from full-length papers through titles, abstracts, paragraphs, diagram texts and find co-occurrence of statistical significance between text corpuses [84]. Some methods present grammatical structures as networks considering properties of semantic notion and analyses with kernel-based methods (mostly an SVM) [69]. Other studies reassemble text corpus to integrate PPI-related information such as phosphorylation, domain interactions, and homology [85,86]. Literature curation is managed by many accessible protein databases such as Yeast Proteome Database (YPD) [87], Database of Interacting Proteins (DIP) [88], BioGRID and HPRD. In addition, there are some TM-based methods/tools that provide multiple-perspective evidence for PPI extraction, such as BioRAT (Biological Research Assistant for Text mining) [89], eFIP (Extracting Functional Impact of Phosphorylation) [85], FACTA (Finding Associated Concepts with Text Analysis) [90] and Hit Predict [86].

## 4. Strategy for Integrative Analysis

Studies in this category make use of a classification algorithm to integrate interaction-related features. With these available physical and non-physical features, classifiers are trained to distinguish between positive and negative examples. It is a challenge to integrate evidence variants in confidence and coverage to increase PPI prediction coverage and accuracy. The common process of PPI prediction by evidence-combining methods includes several steps.

Step 1: Choose appropriate evidence. Evidence must be carefully chosen with content specialized for each different network. Moreover, the following issue must be taken into consideration: Is this evidence a discovery of a global PPI in an unexploited species, or is it a meticulous digging of interaction sites in model species? It should be noted that there is a widespread misconception that “more evidence yields better results”. In a prediction process, blindly incorporating multiple sources of evidence could influence the results and yield other biases [42].

Step 2: Encode protein pairs with evidence. The common encoding process transforms individual or homogeneous evidence into a feature vector representing each pair of proteins. The goal is to convert them to solve the problem of binary classification. These features may represent a particular source of information such as correlations of gene expression, phylogenetic profiles, sequence-based signatures, GO functional annotation and chemical properties. There are many modes to encode evidence sources into a featured vector, to choose statistical standard and data dimensions, and to check the normalization affect or the reliability of different computational predictions [22,45].

Step 3: Different strategies are adopted to merge classifiers into integrative datasets. Some studies use uniform evidence with a similarly encoded rule in one step. Some studies first train datasets with multiple independent evidence and then cross-validate and integrate multiple independent sets of training results to reduce potential bias. Others use single training or integrating probability score to uncover a novel subset of the whole interactome. Many classifiers have been introduced to predict PPIs including, Artificial Neural Network (ANN), Decision Tree (DT), K-Nearest Neighbor (KNN), Logistic Regression (LR), Naive Bayes (NB), Random Forest (RF), Support Vector Machine (SVM), etc. (Table 3).

However, studies of PPIs are diverse in target species, data sources, demand of accuracy and coverage, which are various in details and processes. In this paper, we are focused on introducing several independent strategies for integrative analysis. Some related studies are listed in Table 3.

### 4.1. Exploratory PPI Predictions Using Combinated Vector Descriptors

Some studies encode evidence sources with uniform rule and use high-dimensional concatenated vectors to present information of uniformed evidence.

Case 1: The Multi-Scale Continuous and Discontinuous (MCD) feature method [58] (developed from the auto-covariance (AC) [76] method) captures the interactions from continuous and discontinuous binding patterns present within a protein sequence. MCD divides the entire protein sequence into four strings of equal length. For each string, three types of descriptors (composition, transition and distribution that have evidence based on amino acid sequences) are used to represent amino acid properties. Then, a high-dimensional concatenated vector is used to present information of sector combination (4-bit binary of MCD feature) and encode evidence in a protein pair. At last, minimum redundancy maximum relevance (mRMR) is applied for the feature selection, and the SVM classifier finally performs the prediction tasks.

Case 2: Another method is to predict PPIs using graph invariants and a neural network [57]. It considers the primary structure of domains as a numerical sequence that combines even invariants containing graph invariants derived from graph-theoretic models of individual amino acids (including weighted domination (g), averaged eccentricity (d), circumference (c) and weighted peripheral number (p)), hydrophobicity and charge of each amino acid. Then, vectors train with a neural network to recognize their targets.

### 4.2. Exploratory PPI Predictions Using Probabilistic Classification Scoring

Some studies construct a PPI network using scoring methods based on probabilistic classification decision making. These methods evaluate particular potential of protein interactions through the likelihood of a true positive. Take the following individual cases for example.

Case 1: Naive Bayes strategy is proposed for exploring a model network in specific species which lack protein structural information [18,22]. Available evidence includes genomic and proteomic assembled data, ortholog interaction in model organisms, coexpression profiles and enriched protein−domain pairs, as well as shared functional annotations from Gene Ontology (identified the smallest shared biological process (SSBP) score). The probability combines the evidence sources into a naive Bayes model which involves calculating and identifying the max LR of each pair-based evidence, and then integrating the above results with naive Bayes algorithm and generating final composite likelihood ratio from multiplicative LR.

Case 2: InPrePPI (an integrated evaluation method based on genomic context for predicting protein−protein interactions in prokaryotic genomes) [21] uses AC value (an integrated value of the accuracy and coverage) to integrate data. In this study, each protein pair of three positive datasets (KEGG, EcoCyc, and DIP) is encoded by four methods of phylogenetic profile (PP), gene cluster (GC), gene fusion event (FE) and gene neighbor (GN), respectively. The accuracy and coverage is calculated based on each method. Finally, an integrated score for each protein pair is presented by calculating weight and normalized AC value.

### 4.3. Prediction of Protein–Protein Interaction Sites

Proteins associate with each other through specific binding sites. These protein–protein interaction sites (PPISs) are believed to be good contributors to the recognition of binding residues under specific chemical and physical statuses. Since PPISs mark the central position of interactions and are less efficiently captured by experimental methods, computational approaches have been developed to model the discrimination between interacting and non-interacting sites for prediction of PPIS. Many studies proposed PPI site prediction methods by training with structure-based and sequence-based evidence. Computational approaches for PPI prediction using structural information have gained more attention due to the rapid growth of structural information (in PDB). In this review, the following individual studies are taken as examples.

Case 1: A prediction server of PPIS named PSIVER [64] predicts binding residue protein pairs by using the naive Bayes (NB) classifier and kernel density estimation (KDE) with two distinct features: position-specific scoring matrix (PSSM) and predicted accessibility (PA). Individual classifiers are trained on the basis of PSSM and PA evidence, respectively. Then, results are combined into a score for classifying GSP and GSN.

Case 2: In a study by Dhole et al. (2014), L1-regularized logistic regression (L1-logreg) was developed as a classifier by training evidence based on PSSM, averaged cumulative hydropathy (ACH) and predicted relative solvent accessibility (RSA), which includes evolutionary conservation and chemical/functional information of amino acids [63].

Case 3: The SSWRF method [65] is introduced in order to assemble the SVM and sample-weighted random forest (SWRF). A lower-dimensional vector represents the evidence of the PSSM-derived feature, averaged cumulative hydropathy (ACH) and averaged cumulative relative solvent accessibility (RSA). It processes some vectors of a given training dataset with SVM. The generated scores to evaluate samples and to calculate weights are further utilized for training with SWRF. Finally, the ensemble algorithm of the SVM and SWRF is executed to predict query inputs.

## 5. Performance Evaluation of PPI Prediction

Generally, cross-validation is employed to evaluate the prediction of performance of the proposed method. Some studies evaluate the performance of prediction by cross-validating datasets from different sources (databases, experimental methods or organisms). Some studies randomly divide testing datasets into several equally sized subsets, and each subset is used as a test set [21,65,76].

The following assessments are taken into account to perform evaluation: Precision, Recall (Sensitivity), Specificity, Overall Prediction Accuracy, Matthews’s Correlation Coefficient (MCC), F-measure, Receiver Operating Characteristic (ROC) and Area Under the ROC Curve (AUC). These assessments compute the accuracy and deviation to evaluate the feasibility and robustness of a PPI prediction method. Some are defined as follows:

Precision = TP/(TP + FP)(1)

Recall = TP/(TP + FN)(2)

Specificity = TN/(FP + TN)(3)

Overall Prediction Accuracy = (TP + TN)/(TP + TN + FP + FN)(4)

(5) $$\mathrm{MCC}=\frac{\left(\mathrm{TP}\times \mathrm{TN}\right)-\left(\mathrm{FP}\times \mathrm{FN}\right)}{\sqrt{\left(\mathrm{TP}+\mathrm{FP}\right)\times \left(\mathrm{TP}+\mathrm{FN}\right)\times \;\left(\mathrm{TN}+\mathrm{FP}\right)\times \left(\mathrm{TN}+\mathrm{FN}\right)}}$$

F-measure = 2 × (Recall × Precision)/(Recall + Precision) = 2TP/(2TP + FP + FN)(6)

TP (true positive) is the number of the predicted PPIs found in the GSP; FP (false positive) is the number of the predicted PPIs not found in GSP; FN (false negative) is the number of PPIs in the GSP that failed to be predicted by the method false positive; TN (true negative) is the number of true non-interacting pairs predicted correctly. MCC, F-measure, ROC and AUC are important assessments. MCC is a measure of the quality of binary classification, which is a correlation coefficient between the observed and predicted results (it returns a value between −1 and +1. MCC equal to 0 is regarded as a completely random prediction, −1 is regarded as a completely wrong prediction and 1 is regarded as a perfect prediction). F-measure is the harmonic mean of Recall and Precision which combines Recall and Precision with balanced weights. In addition, ROC curve and AUC value illustrate performance of a binary classifier system by graphical plot. ROC curve is generated by plotting the TP rate against the (FP rate at various thresholds, and AUC values are used for comparison between methods.

## 6. Conclusions

Biology relies on the concerted actions of proteins organized in networks. The role of computational biology research in the area of protein–protein interaction prediction methodologies has recently gained widespread attention. Many tools have been developed to facilitate system biologists, not only in PPI prediction but also in defining their binding residues involved at interaction interfaces. In this review, we presented workflows to predict large-scale PPIs through a variety of evidence methods. However, the result of “interactions” is solely a definition of compatibility between two proteins with respect to evolution, function and structure, regardless of their relative reactivity.

There is still much space for further improvements to reach realistic interactions. For this purpose, high quantity and quality datasets are indispensable. The significant increase in the prediction coverage and accuracy during the past several years is mainly caused by the accumulation of credible data from genome sequencing, PPI experimental detection and protein 3D structure definition. It can be anticipated that, with more and more information available in the future, the prediction potential will be improved and the corresponding combined methods will acquire better performance. On the other hand, more precise methods are also required in this regard. More time is needed for the development of even more powerful machine learning methods (like deep neural networks), along with the systemic understanding of the essential mechanism of PPIs. We hope that the present work will inspire PPI predictors toward further evaluation and improvements.

## Figures and Tables

**Table 1:** Interaction databases for construction of gold standard (until September 2016).

Name	Description	Points/Edges	C	E	L	O	Last Update	Ref. I	Ref. II
BioGrid 3.4	An interaction repository with data compiled through comprehensive curation efforts.	65,099/836,212	N	P	P	61	September 2016	http://thebiogrid.org/	[24]
IntAct 4.2.5	Provides a strong, freely available, open source database system and analysis tools for molecular interaction data.	93,856/653,104	N	P	P	8	September 2016	http://www.ebi.ac.uk/intact/	[25]
PDB	A database containing experimentally determined three-dimensional structures of proteins.	126,079/NA	N	P	P	NA	September 2016	http://www.wwpdb.org/	[36]
STRING	A database including protein interactions containing both physical and functional associations.	9.6 million/184 million	P	P	P	2031	September 2016	http://string-db.org/	[26]
APID	Based on known experimentally validated PPIs and integrated interactomes with a methodological approach to report quality levels and coverage over the proteomes.	90,379/678,441	N	P	P	25	June 2016	http://bioinfow.dep.usal.es/apid/	[27]
DIP	A database combining experimental PPI information from a variety of sources.	28,764/81,627	N	P	P	826	Febrary 2014	http://dip.doe-mbi.ucla.edu/dip/	[28]
HitPredict 4	A resource of experimentally determined PPI with reliability scores.	70,808/398,696	N	P	N	105	September 2015	http://hintdb.hgc.jp/htp/	[29]
MINT	Focuses on experimentally verified protein−protein interactions mined from the scientific literature by expert curators.	25,530/125,464	N	P	P	611	September 2013	http://mint.bio.uniroma2.it/mint/	[30]
TAIR-nbrowse	Provide Arabidopsis PPI data curated from the literature by TAIR curators.	2452/8626	N	P	P	1	September 2011	http://www.arabidopsis.org/tools/nbrowse.jsp	[31]
HPRD Release 9	A centralized platform to integrate interaction networks of human protein.	30,047/41,327	N	P	N	1	April 2010	http://hprd.org/	[32]
PINA2.0	An integrated platform for protein interaction network construction, filtering, analysis, visualization and management.	12,969/365,930	N	P	N	7	May 2014	http://cbg.garvan.unsw.edu.au/pina/	[33]
Negatome 2.0 *	A collection containing experimentally supported non-interacting protein pairs and domain pairs which are unlikely engaged in direct physical interactions.	3376/6532	N	P	P	NA	2014	http://mips.helmholtz-muenchen.de/proj/ppi/negatome/	[37]

Points/Edges, number of interactors (proteins)/number of interactions; C, computationally supported; E, experimentally supported; L, Literature curated; O, number of organisms; Ref., reference resources; N, negative PPIs contained; P, positive PPIs contained; *, negative datasets of PPIs; NA, Not Available.

**Table 2:** Annotated protein pairs with diverse evidence.

Categories	Feature	Abbreviation	Ref.
EVO	Gene Fusion Event	FE	[21,22,45,46,47,48]
	Gene Cluster	GCL	[21]
	Gene Neighborhood	GN	[21,22,45,47]
	Pylogenetic Profile	PP	[16,21,22,45,46,47,49,50]
FF	GO Cellular Component	COM	[1,16,22,45,46,50,51,52]
	Coessentiality	ESS	[45,50]
	Gene/Protein Coexpression	Exp	[1,16,18,22,45,46,47,50,52,53,54]
	GO Molecular Function	FUN	[1,16,22,45,46,48,50,52]
	Colocalization	Loc	[47,53,54]
	Ortholog/ Sequence Similar	ORT	[1,18,22,35,45,50,51,52,53,54,55,56]
	GO Biological Process	PRO	[1,16,18,22,45,46,47,48,50,51,52]
	Coregulation/Transcriptional Regulation	Reg	[45]
TOP	Graphical Invariants	GI	[57]
	Probabilistic Graphical Model	PGM	[57]
	Small-World Clustering Coefficients	SCC	[20,58]
SEQ	Conjoint Triad	COT	[35,58,59]
	N-grams	NGR	[60,61]
	ORF Codon Usage	ORF	[62]
	Position-Specific Scoring Matrix	PSSM	[63,64,65]
	2D Structure	2DS	[20]
STR	3D Structure	3DS	[46,66,67]
	Average of the Cumulative Hydropathy Indices	ACH	[63,65]
	Domain–Domain Interaction	DDI	[1,16,18,22,35,45,46,47,48,54]
	DSSP Structure in PDB	DSSP	[20]
	Electrostatics	ELE	[68]
	Protein Fold	Fold	[47]
	Generalized Born	GB	[68]
	High Quality AA Indices	HQI	[20]
	Predicted Accessibility	pA	[64]
	Physico-Chemical Properties	PHC	[20,57,66,67]
	PSIPRED Structure	PSIP	[1,20]
	Posttranslational Modifications	PTM	[1,54,68]
	Relative Solvent Accessibility	RSA	[56,63,65]
	Surface Area	SA	[68]
	Van Der Waals Forces	VDW	[68]
TM	Literature-Curated	LC	[69]

Categories: Evolutionary relationship (EVO), Functional features (FF), Network topological (TOP), Sequence-based signatures (SEQ), Structure-based signatures (STR), Text mining (TM). The definitions of abbreviations are based on references and customizations.

**Table 3:** Some studies or online tools of PPI prediction by evidence-combining methods (until September 2016).

Class	Description	Classifiers	Evidence	Organisms	Ref.	(URL) (Last Update) (Points/Edges)
DDI	iPfam: catalogs of protein family interactions, including domain and ligand interactions, calculated from known structures	NA	PHC, 3DS	NA	[66]	(http://ipfam.org/) (June 2013) (>9500/15,500)
DDI	3did: database of three-dimensional interacting domains is a collection of DDIs in proteins for which high-resolution known 3D structures	NA	PHC, 3DS	NA	[67]	(http://3did.irbbarcelona.org/) (June 2016) (648/9952)
DDI	DOMINE is a database of known and predicted DDIs	POI	Exp, PP, FE, FUN, PRO, COM, DDI, 3DS	NA	[46,82]	(http://domine.utdallas.edu/cgi-bin/Domine) (September 2010) (5410/26,219)
DDI	Combine protein interaction datasets from multiple species to construct DDIs	NB, EC	FUN, PRO, GF, DDI, etc.	4 (*Hs*, *Dm*, *Sc*, *Ce*)	[48]	NA
PPI	Predicting PPIs in *Arabidopsis thaliana*	EC	ORT, COM, PRO	1 (*At*)	[51]	NA
PPI	CitrusNet: sweet orange PPI network	KNN	DDI, ORT, COT	1 (*Cs*)	[35]	(http://citrus.hzau.edu.cn/orange/ppi/index.php) (June 2013) (8,195/124,491)
PPI	A predicted interactome for *Arabidopsis*.	EC	Exp, ORT, Loc	1 *(At*)	[53]	NA
PPI	PRIN: a predicted rice interactome network	EC	FUN, PRO, COM, Exp, ORT	1 (*Os*)	[52]	(http://bis.zju.edu.cn/prin/) (2010) (5049/76,585)
PPI	TSEMA: predicts the interaction between two families of proteins based on Monte Carlo approach	MC	PP	NA	[49]	(http://tsema.bioinfo.cnio.es/)
PPI	Predicting PPI using graph invariants and a neural network	NN	PGM, GI, PHC	NA	[57]	NA
PPI	IID: integrated interactions database providing tissue-specific PPIs for model organisms	EC	Exp, ORT, etc.	6 (*Sc*, *Ce*, *Dm*, *Mm*, *Hs*, *Rn*)	[1]	(http://dcv.uhnres.utoronto.ca/iid/) (March 2016) (NA/1,741,568)
PPI	FpClass: interactions and properties of human proteins	association analysis	DDI, FUN, PRO, COM, PTM, Exp, ORT, PSIP	1 (*Hs*)	[1]	(http://ophid.utoronto.ca/fpclass/) (NA) (10,531/250,498)
PPI	PAIR: the predicted Arabidopsis interactome resource	SVM	PP, PRO, FUN, COM, Exp, DDI	1 (*At*)	[16]	(http://www.cls.zju.edu.cn/pair/)
PPI	SPPS: sequence-based protein partners search	SVM	COT	5 (*Sc*, *Ce*, *Dm*, *Ec*, *Hs*)	[59]	(http://mdl.shsmu.edu.cn/SPPS/) (November 2011) (Hs = 23,719/39,191; Mm = 16,542/1225; Ce = 5348/4973; Dm = 8921/22,482; Sc = 16,506/25,064)
PPI	PIPs: human PPI prediction database	NB	Exp, ORT, DDI, Loc, PTM	5 (*Sc*, *Ce*, *Dm*, *Ec*, *Hs*)	[54]	(http://www.compbio.dundee.ac.uk/www-pips/) (September 2008) (NA/79441)
PPI	Six classifiers and different biological data were used to predict interactions	RF, kRF, NB, DT, LR, SVM	Exp, FUN, PRO, COM, ESS, Reg, FE, GN, PP, ORT, DDI, etc.	NA	[45]	NA
PPI	SSWRF: an ensemble of SVM and SWRF method	SVM, SWRF	PSSM, ACH, RSA	NA	[65]	NA
PPI	Sequence-based approach is developed by combining MCD and SVM methods	MCD, SVM	COT, SeqS	1 (*Sc*)	[58]	NA
PPI	PrePPI: predicts PPI using both structural and nonstructural information	LR	ORT, FUN, PRO, COM, ESS, Exp, PP, etc.	2 (*Sc*, *Hs*)	[50,91]	(http://bhapp.c2b2.columbia.edu/PrePPI/) (August 2011) (Sc = NA/31,402; Hs = NA/317,813)
PPI	MLPPI: multi-level machine learning prediction of PPI in yeast	SVM	2DS&PHC (PSIP, DSSP, HQI), SEQ, etc.	1(*Sc*)	[20]	(http://zubekj.github.io/mlppi/) (NA) (NA)
PPI	Probabilistic model of the human PPI network	NB	PRO, Exp, ORT, DDI	1 (*Hs*)	[18]	NA
PPI	Characterization and prediction of PPI in the yeast	LR	DDI, Fold, FE, PP, GN, Loc, PRO, Exp	1 (*Sc*)	[47]	NA
PPI	InPrePPI method: an integrated method for prediction of PPI	AC	GCL, PP, FE, GN	1 (*Ec*)	[21]	(http://inpreppi.biosino.org/InPrePPI/index.jsp) (NA) (6,429/17,855)
PPI	Global genome-scale PPI network in *Arabidopsis thaliana.*	NB	ORT, FE, GN, PP, FUN, PRO, COM, Exp, DDI	1 (*At*)	[22]	NA
PPIS	LORIS method: sequence-based L1-logreg classifier proposed to identify PPIS	L1-logreg	PSSM, ACH, RSA	NA	[63]	NA
PPIS	Struct2Net, iWRAP & Coev2Net	PGM, LR, etc.	ORT	3 (*Hs*, *Sc*, *Dm*)	[55]	(http://groups.csail.mit.edu/cb/struct2net/webserver) (2012) (NA)
PPIS	PRISM2: protein interactions by structural matching	EC	RSA, ORT	NA	[56]	(http://cosbi.ku.edu.tr/prism/) (NA) (NA)
PPIS	MIEC-SVM: structure-based method for predicting protein recognition specificity	SVM	VDW, ELE, GB, SA, PTM, etc.	NA	[68]	(http://wanglab.ucsd.edu/MIEC-SVM/) (NA) (NA)
PPIS	PSIVER method	NB, KDE	PSSM, pA	NA	[64]	NA

Points/Edges, number of interactors (proteins or domains)/number of interactions; Ref., reference resources; NA, Not Available; URL, Uniform Resource Locator (some sites are currently under maintenance); DDI, Domain–Domain Interaction; PPI, Protein–Protein Interaction; PPIS, Protein–Protein Interaction Site. Classifiers: AC, Integrated Value of the Accuracy and Coverage; ANN, Artificial Neural Network; DT, Decision Tree; EC, Evidence Counting; KDE, Kernel Density Estimation; KNN, K-nearest Neighbor; kRF, RF similarity-based k-Nearest-Neighbor; L1-logreg, L1-regularized Logistic Regression; LR, Logistic Regression; MC, Monte Carlo; MCD, Multi-scale Continuous and Discontinuous Sequence Representation Approach; NB, Naive Bayes; PGM, Probabilistic Graphical Model; POI, Prediction Overlap Index; RF, Random Forest; SVM, Support Vector Machine; SWRF, Sample-weighted Random Forest. Organisms: *At*, *Arabidopsis thaliana*; *Ce*, *Caenorhabditiselegans*; *Cs*, *Citrus sinensis*; *Dm*, *Drosophila melanogaster*; *Ec*, *Escherichia coli*; *Hs*, *Homo sapiens*; *Mm*, *Musmusculus*; *Os*, *Oryza sativa*; *Rn*, *Rattusnorvegicus*; *Sc*, *Saccharomyces cerevisiae*.
